# Complete Genome Sequence of Lumpy Skin Disease Virus Isolate SERBIA/Bujanovac/2016, Detected during an Outbreak in the Balkan Area

**DOI:** 10.1128/genomeA.00882-17

**Published:** 2017-08-31

**Authors:** Ivan Toplak, Tamaš Petrović, Dejan Vidanović, Sava Lazić, Milanko Šekler, Marija Manić, Miloš Petrović, Urška Kuhar

**Affiliations:** a Institute of Microbiology and Parasitology, Veterinary Faculty, University of Ljubljana, Ljubljana, Slovenia; b Virology Department, Scientific Veterinary Institute Novi Sad, Novi Sad, Serbia; c Department for Laboratory Diagnostics, Veterinary Specialized Institute Kraljevo, Kraljevo, Serbia; d Veterinary Specialized Institute Niš, Niš, Serbia

## Abstract

The lumpy skin disease virus (LSDV) isolate SERBIA/Bujanovac/2016 consists of 150,661 nucleotides and has a 99.95% nucleotide identity with the Neethling Warmbaths LW strain isolated in South Africa in 1999. This is the first complete LSDV genome determined in Serbia and also in the Balkan area.

## GENOME ANNOUNCEMENT

Historically, the lumpy skin disease virus (LSDV) was restricted to Africa, but in 2013 and 2014, outbreaks were reported in several Middle Eastern countries (1). Since 2013, the disease has been present in Turkey (2). In August 2015, the first incursion of LSDV was reported in Greece and Cyprus, and since 2016, LSDV has been rapidly spreading into six Balkan countries (Bulgaria, FYR Macedonia, Serbia, Kosovo, Montenegro, and Albania), causing serious challenges to the implementation of successful control measures (3, 4; see https://www.oie.int/wahis_2/public/wahid.php/Diseaseinformation/WI).

The first clinical suspicion of LSDV in Serbia was on 4 June 2016 in the settlement of Liljance in the municipality of Bujanovac. The LSDV outbreak was characterized by fever and nodules on the skin, mucous membranes, and internal organs. The virus was isolated on MDBK cell culture, and its complete genome was determined by next-generation sequencing using Ion Torrent technology.

Total DNA was extracted with the QIAamp DNA minikit (Qiagen, Hilden, Germany) and fragmented with Covaris M220. A library was prepared with the GeneRead DNA Library L core kit (Qiagen) and sequenced on the Ion PGM platform at the Veterinary Faculty, University of Ljubljana, Slovenia.

For genome assembly, reads were mapped to the reference genome (GenBank accession no. AF409137) using the Geneious software (Biomatters Ltd., Auckland, New Zealand). The consensus sequence was aligned with the previously published LSDV genomes using MAFFT (5). For resolving discrepancies that were identified after manual inspection of the genome reference mapping, 19 genome segments were sequenced by Sanger sequencing (6). Open reading frames were predicted with the Genome Annotation Transfer Utility (GATU) (7) relative to other LSDV genomes.

A total of 39,296 reads were mapped to the assembled LSDV SERBIA/Bujanovac/2016 genome, with an average coverage depth of 43.4× and an average map length of 163 nucleotides (nt). The genome consists of 150,661 nt, with a central coding region of 156 protein-coding genes flanked by two inverted terminal repeat (ITR) regions of at least 2,357 bp at both terminal parts.

The alignment of the SERBIA/Bujanovac/2016 genome with the complete LSDV genomes available in GenBank revealed that it shares 99.95% and 99.99% nucleotide identities with the virulent South African Neethling Warmbaths LW strain (AF409137) (8) and the Evros/GR/15 strain (KY829023), respectively. The Serbian LSDV genome differs from the South African strain in 12 amino acid modifications (D/N in gene LD005, E/K in gene LD006, H/R in gene LD017, C/F in gene LD059, K/E in gene LD087, S/L in gene LD094, K/deletion in gene LD096, E/K and M/I in gene LD126, S/F in gene LD128, M/I in gene LD139, and R/K in gene LD148), 11 nucleotide changes that do not have an effect on the coding region, and 8 nucleotide changes in the intergenic regions. A 15-nt deletion (TAAGTGGAAGCCAAT) in the terminal noncoding part of the ITR region of the Serbian LSDV strain was identified. The same 15-nt deletion was also found in the LSDV isolate Evros/GR/15, suggesting possible stability of this deletion in Balkan field samples for at least 1 year.

### Accession number(s).

The complete genome sequence of the strain SERBIA/Bujanovac/2016 has been deposited in GenBank under accession number KY702007.
